# Innovative Application of Nanomaterials in Vegetable Cultivation: Recent Advances in Growth Promotion and Stress Tolerance

**DOI:** 10.3390/nano15211659

**Published:** 2025-10-31

**Authors:** Wenxuan Lv, Yixue Bai, Dongyang Zhu, Changzheng He, Fengjiao Bu, Yusong Luo, Ping Zhao, Yanhong Qiu, Zunzheng Wei, Jie Zhang, Shaogui Guo, Yongtao Yu, Jingfang Wang, Yi Ren, Guoyi Gong, Haiying Zhang, Yong Xu, Guang Liu, Sihui Dai, Maoying Li

**Affiliations:** 1 State Key Laboratory of Vegetable Biobreeding, National Engineering Research Center for Vegetables, Beijing Key Laboratory of Vegetable Germplasms Improvement, Beijing Vegetable Research Center, Beijing Academy of Agriculture and Forestry Science, Beijing 100097, China; lwx2656478686@163.com (W.L.); 18811702979@163.com (Y.B.);; 2 College of Horticulture, Hunan Agricultural University, Changsha 410128, China; 3 Yuelu Mountain Laboratory, Changsha 410082, China; 4 Changle County Agriculture and Rural Bureau, Fuzhou 262400, China; 5 Institute of Grassland, Flowers and Ecology, Beijing Academy of Agriculture and Forestry Sciences, Beijing 100097, China; 6 Institute of Vegetable Crop, Jiangsu Academy of Agricultural Sciences, Nanjing 210014, China

**Keywords:** nanomaterials, vegetable cultivation, growth promotion, stress tolerance

## Abstract

Vegetables are crucial to human diet and health. To ensure sustainable vegetable production, regulatory measures are needed to enhance seed germination, plant growth, and resilience to extreme environmental conditions. Nanomaterials (NMs), owing to their high surface area, nanoscale dimensions, and unique photocatalytic properties, exhibit remarkable biological effects, such as promoting germination and growth, as well as improving stress resistance in crops, offering novel solutions to key challenges in vegetable cultivation. This review summarizes the absorption pathways of NMs in plants, specifically through the leaves and roots of vegetables. Their uptake and translocation occur via passive diffusion, active transport, and endocytosis, with key influencing factors including particle size, chemical composition, surface charge, and surface modifications. We further evaluate the advantages of nanofertilizers and nanopesticides, in vegetable production over their traditional counterparts, focusing on improvements in seed germination rates, seedling vigor, biotic and abiotic stress tolerance, and overall yield and quality. Through this review, we aim to offer comprehensive insights into the application of NMs in vegetable crop production.

## 1. Introduction

Vegetables are essential components of the human diet, providing diverse nutrients for maintaining normal physiological functions [1]. Vegetable cultivation is characterized by short growth cycles, high productivity, and strong adaptability. However, it encounters significant challenges, including soil pollution, pesticide residues, poor germination efficiency, and frequent stresses [2]. These issues not only reduce crop yield and quality but also pose profound and enduring risks to both the environment and human health [3,4]. Nanomaterials (NMs), as an emerging agricultural technology, present a promising solution to these challenges.

NMs are defined as materials with at least one dimension in the nanoscale range (1–100 nm) or structures composed of nanoscale units, corresponding to approximately 10–1000 closely packed atoms [5,6]. Compared to conventional materials, NMs exhibit superior physical, chemical, and biological properties due to their high surface-to-volume ratio, small size, and unique photocatalytic activity [7,8,9,10]. These exceptional characteristics have enabled their widespread application in diverse fields, including biosensing, water purification, photocatalysis, and antibacterial agents [8]. Additionally, NMs have garnered significant attention for their capacity to modulate the adaptation responses of plants to various biotic and abiotic stresses [8]. In vegetable production systems, NMs are increasingly being utilized to enhance seed germination efficiency and seedling vigor, mitigate stress impacts, and improve crop yield and quality [6].

This review systematically examines the application of nanotechnology in vegetable cultivation, with particular emphasis on seed germination enhancement, plant growth promotion, and stress mitigation under adverse environmental conditions. Notably, as we embrace the potential benefits of NMs in vegetable cultivation, it is imperative to address certain concerns. The phytotoxicity brought about by excessive amounts of NMs takes a toll on seed germination and biomass production, disrupts the photosynthesis system, induces oxidative stress, compromises cell membrane integrity, alters gene expression, causes DNA damage, and leads to epigenetic variations in plants [11,12]. Given that vegetables are consumed fresh and in substantial quantities, evaluating the accumulation and potential toxicity of NMs in edible tissues, especially in leafy or root vegetables, becomes of paramount importance, as any adverse effects could have far-reaching consequences for public health. Therefore, we discuss current challenges and future perspectives to guide forthcoming research in this field.

## 2. Agricultural Applications of NMs in Vegetable Cultivation

**Figure 1:**
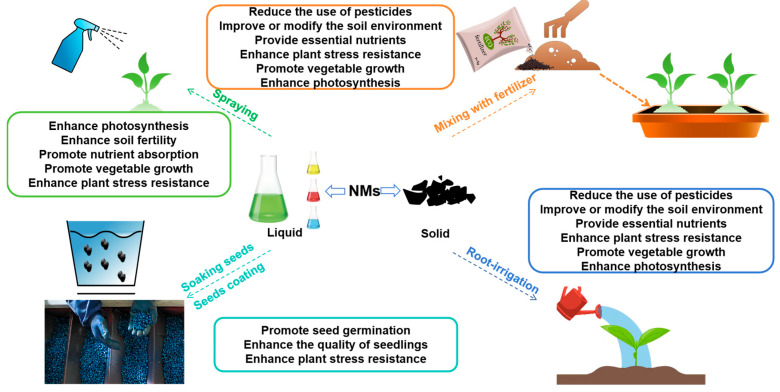
Applications and roles of NMs in agriculture. NMs are used through various application methods in agricultural practices, such as foliar spraying, seed priming, seed coating, root irrigation, and fertilizer incorporation to enhance crop yield and improve product quality.

A series of products with nano-features, namely nanofertilizers, nanopesticides, nanoanimal feed supplements, and nano-based devices and sensors, have been extensively employed in diverse agricultural domains. Of these applications, nanofertilizers and nanopesticides have gained particular prominence in commercial agriculture owing to their proven effectiveness in mitigating critical agricultural challenges, such as low nutrient use efficiency, excessive pesticide application, and environmental pollution (Figure 1) [13].

### 2.1. Nanofertilizers

Nanofertilizers represent a class of NMs that function either as direct plant nutrients (macro- and micronutrients) or as nutrient carriers [14]. With their nanoscale dimensions and high surface area-to-volume ratio [15], nanofertilizers facilitate controlled nutrient release and efficient plant uptake, thereby reducing fertilizer requirements and minimizing nutrient losses via runoff and leaching. Thus, compared to conventional fertilizers, nanofertilizers are superior in nutrient utilization efficiency, application cost, and environmental impact.

A key advantage of nanofertilizers is their tunable release kinetics, which enables sustained nutrient delivery to plants over prolonged periods [16]. This controlled-release manner maintains optimal soil nutrient levels without frequent fertilization [17]. Various nanofertilizers have demonstrated this feature, including ZnO [18], CeO [19], Se [20,21], TiO_2_ [22,23], Graphene [24], Chitosan/ZnO [25], Fe_3_O_4_ [26,27], Mn_3_O_4_ [28], etc. (Table 1). When applied, their controlled release enhances seed germination, improves water uptake, and promotes biomass accumulation. Additionally, TiO_2_ [22] and Si [29] NMs have been shown to improve soil structure, enhance water retention, and boost biotic stress resistance in plants. Moreover, these NMs mitigate phytotoxicity by reducing reactive oxygen species (ROS) accumulation and preserving photosynthetic apparatus from oxidative damage [30].

In summary, nanofertilizers demonstrate multifaceted benefits for modern agriculture by significantly enhancing nutrient use efficiency, substantially reducing fertilizer input requirements, and improving soil physicochemical properties. These combined effects promote robust seed germination, vigorous plant growth, and strengthened stress tolerance. Nanofertilizers represent a revolutionary approach to plant nutrition, enabling more efficient, sustainable, and tunable nutrient delivery systems.

### 2.2. Nanopesticides

Vegetable crops face persistent threats from both biotic and abiotic stressors [45]. While conventional pesticides provide partial protection, their detrimental ecological impacts have raised significant environmental concerns [46]. Nanopesticides, engineered through nanotechnology with controlled size distributions, offer enhanced efficacy and reduced environmental impact, representing an advanced alternative to traditional pesticides for agricultural plant protection [47]. These nanoformulations demonstrate superiority over traditional pesticides through enhanced water solubility and bioavailability of active ingredients, coupled with improved protection against environmental degradation. Nanopesticides also represent an innovative strategy for crop protection against pathogens, weeds, and pests [48].

Numerous NMs have demonstrated efficacy in plant protection, including CuO [49,50,51,52], MgO [53],Chitosan-decorated copper oxide (CH@CuO) [54], La_2_O_3_ [55], Ag [56], S [57], CoFe_2_O_4_ [58], NiFe_2_O_4_ [58], etc. (Table 2). They either protect plants from pathogen infection and/or enhance plant disease resistance through various mechanisms, such as disrupting hyphal cell walls, inhibiting hyphal growth, and preventing spore germination [59]. NMs like (GO) [60], The CeO_2_-based nanohybrid (MON@CeO) [61], and Ag [62] rapidly penetrate the pest cuticle to induce toxicity and impair reproductive capacity. In addition, certain nanopesticides can activate plant defense pathways, providing indirect protection against pests and pathogens [63]. For instance, Chitin nanofiber (CNF) [64], Cu [65], Carbon [66], Graphene-Cu [67], Bio-Mn [68] can systematically induce stress tolerance in vegetable plants, thereby enhancing their overall resilience to pests and diseases.

Collectively, nanopesticides are increasingly influencing conventional agricultural practices and advancing sustainable plant protection strategies. They directly suppress pest/pathogen infection and/or indirectly enhance plants’ stress tolerance through nanomaterial-specific properties [69].

**Table 2:** Nanopesticides applied in vegetable cultivation.

Nanopesticides	Concentration	Size (nm)	Vegetable Species	References
Fe_3_O_4_	10 mg/L	N/A	Coriander	[26]
Ag	1 g	415 nm	Carrot	[56]
Ag	N/A	43–74 nm	N/A	[62]
Cu	1 g	339 nm	Carrot	[56]
Graphene-Cu	100 mg/L	22–97 nm	Tomato	[67]
CH@CuO	100 mg/L	32.74 ± 2.3 nm	Tomato	[54]
S	100 mg/L	N/A	Tomato	[57]
CoFe_2_O_4_ and NiFe_2_O_4_	500 ppm	25 nm	Pepper	[58]
CuO	3 mg/mL	3.59–6.05 nm	Potato	[53]
MgO	3 mg/mL	3.71–6.58 nm	Potato	[53]
Fe_2_O_3_ NM-B, NM-H	0.25 mM, 0.125 mM	23.45 nm, 15.8 nm	Cucumber	[70]
La_2_O_3_	500 mg/L	19.93 ± 6.95 nm	Cucumber	[55]
Cu_3_(PO_4_)_2_·3H_2_O	10 mg/L	N/A	Watermelon	[71]
Bio-Mn	100 µg/mL	27.0–65.7 nm	Watermelon	[68]
Cu(OH)	2.5 mg	N/A	Cucumber	[72]
GO	1000 ppm	100 nm	N/A	[60]
S	100 mg/L	70–150 nm	Lettuce	[73]
CNF	1 mg/mL	10–20 nm	Cabbage	[64]
Biochar-iron	500 mg/kg	N/A	Chinese cabbage	[31]
Mn_3_O_4_	N/A	9.7 ± 1.1 nm	Rape	[74]
CeO_2_	N/A	6.8 ± 0.7 nm	Rape	[74]
MON@CeO	200 mg/L	45.4 nm	N/A	[61]
Glycine betain-ZnO	100 mg/L	17.34 nm	Coriander	[75]
CMC-Nar (Naringenin based nanocomposites)	N/A	65 nm	Cumin	[76]
ZnO	50 mg/L	N/A	Tomato	[18]
Cu	400 mg/kg	40 nm	Cucumber	[65]
Bio-Mn	100 µg/mL	27.0–65.7 nm	Watermelon	[68]
Mn_3_O_4_	N/A	8.9 ± 1.4 nm	Cucumber, Lettuce	[28]
SiO_2_	600 mg/L	N/A	Lilium	[77]

## 3. Uptake and Transportation of NMs in Plants

**Figure 2:**
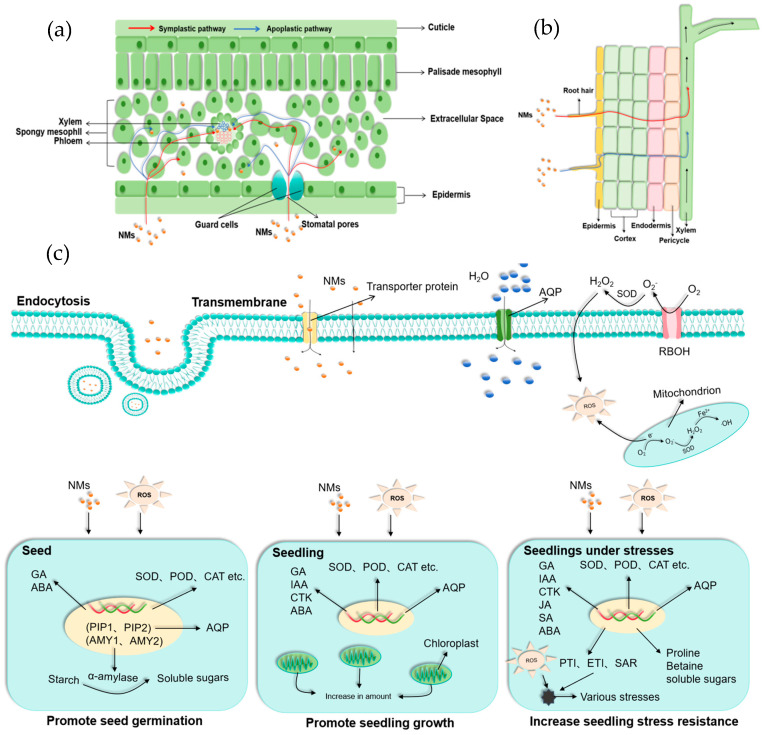
Interaction of NMs with vegetables. (**a**) Vegetable leaves absorb NMs via symplastic and apoplastic routes: NMs penetrate cuticle and palisade mesophyll into extracellular space, may enter through stomata (regulated by guard cells), and interact with spongy mesophyll and phloem. (**b**) Roots take up NMs through root hairs and epidermis into cortex, then to xylem and phloem for systemic movement, a key path for NMs to affect plant physiology. (**c**) NMs may enter cells through passive transport, active transport, and endocytosis. During this process, ROS can be generated. The generated ROS, along with the nanomaterials, jointly influence seed germination (via gibberellin GA, abscisic acid ABA, and α-amylase), seedling growth (via hormones and antioxidant enzymes), and stress resistance (via hormones, enzymes, and osmoprotectants such as proline, betaine, and sugars).

Plants internalize NMs primarily through leaves and roots [78]. NMs are commonly applied in powdered or liquid formulations [79]. To maximize delivery efficiency, powdered NMs are typically dissolved in solvent for precise concentration control, while liquid formulations are applied through targeted methods such as foliar spraying, stem injection, and root drip irrigation. This liquid-phase application significantly increases the interfacial contact between NMs and plant tissues, thereby enhancing absorption efficiency [79]. Notably, the physicochemical properties of NMs, including particle size, chemical composition, surface charge, and functional modifications, directly influence their permeation mode within plant intercellular spaces and transmembrane transport rates. Although conclusive experimental evidence clarifying their specific transport pathways remains lacking, passive diffusion, active transport, and endocytosis are widely recognized as potential primary mechanisms for NMs internalization into plant cells [80] (Figure 2c). These uptake mechanisms play a crucial role in determining how NMs interact with plant tissues, ultimately influencing their potential biological effects and agricultural applicability.

### 3.1. Pathways for Foliar Uptake

Vegetable leaves absorb NMs primarily through stomata and cuticular permeation (Figure 2a) [81]. The uptake mechanism is size-dependent: NMs < 5 nm in diameter can traverse the cuticle via polar channels (0.6–4.8 nm pores) for hydrophilic particles or nonpolar pathways for hydrophobic particles; while larger NMs (>10 nm) bypass cuticular restrictions by entering through stomatal apertures [82,83]. Following foliar entry, NMs initially transfer from the cell wall surface to the plasma membrane via ectodesmata. Subsequent intercellular transport occurs through plasmodesmata, enabling long-distance systemic distribution via the phloem [84].

NMs are intracellularly transported primarily through both passive diffusion and active transport pathways. Smaller NMs typically cross the plasma membrane via passive diffusion, while larger or chemically specific NMs require energy-dependent active transport mediated by membrane transport proteins, a process involving complex systems such as ion channels (e.g., OsGORK, OsAKT1), carrier proteins (e.g., OsHAK1, OsHAK5, OsHAK21), and ion pumps [85]. Additionally, studies have revealed that endocytosis may act as a significant complementary uptake pathway, in which NMs are internalized through membrane-bound vesicles [86].

### 3.2. Pathways for Root Uptake

NMs absorption in plant roots occurs primarily in the unlignified root hair zone (Figure 2b), where mucilage secretion and organic acid exudation contribute to the formation of a negatively charged rhizosphere. Such an electrostatic microenvironment favors the adsorption of positively charged nanoparticles, increasing their bioavailability [78,84,87]. Smaller NMs (3–5 nm) enter plant roots through osmotic pressure, capillary forces, or direct uptake via root epidermal cells [88,89]; while larger NMs (>5 nm) may induce localized membrane destabilization to generate transient pores in epidermal cell walls for entry [83]. Following their entry, internalization occurs through several coordinated mechanisms: (1) ion mimicry via specific channels such as aquaporins or K^+^ transporters, (2) vesicle-mediated endocytosis of surface-functionalized NMs, and (3) protein-assisted transport through membrane carriers like organic acid transporters. These pathways collectively facilitate NM systematic distribution within plant tissues [78].

## 4. The Roles of NMs in Plant Growth and Development

### 4.1. NMs Promote Seed Germination

Seed pretreatment regulates germination by inducing biochemical and physiological changes in seeds. Seed pretreatment with NMs has emerged as an innovative strategy to enhance seed vigor by modulating specific metabolic pathways [90]. Nanopriming technology demonstrates significant improvements in germination rates and synchrony, as well as seedling establishment (Table 3). Although the underlying mechanisms remain incompletely understood, current research has revealed potential modes of action across multiple levels: nanoparticles physically enhance seed imbibition efficiency; biochemically, they activate antioxidant enzyme systems and accelerate starch decomposition for optimized energy supply during seed germination; NMs also adopt physiological regulation by modulating hormonal balance (e.g., gibberellin/abscisic acid) to break seed dormancy; ecologically, NMs regulate rhizosphere microbiome to create favorable growth conditions for seedlings [91,92] (Figure 2c).

Optimal water uptake is essential for initiating metabolic activity and supporting early growth during seed germination [93]. Nanopriming has been reported to accelerate water absorption and prompt earlier germ root processes compared to conventional seed treatment methods [94]. Aquaporins (AQPs), which belong to the major intrinsic protein superfamily, play a crucial role in water homeostasis in vegetables. Some NMs, such as Halloysite Nanotubes (HNTs) and Carbon Nanotubes (CNTs), have been found to function as AQPs and promote water and gas absorption dependent on their unique tubular structure [95,96]. Furthermore, some NMs can stimulate the expression of plasma membrane intrinsic protein 1 and 2 (*PIP1* and *PIP2*) genes, facilitating the formation of water channels on the cell membrane, thereby promoting water influx and improving overall seed germination performance. Additionally, the application of hydrophilic NMs, such as Si-based NMs, to seeds enhances the hydrophilicity of the surrounding environment, thereby increasing seed accessibility to water, which, consequently, accelerates seed germination and boosts seedling vigor [36].

**Table 3:** The effect of NMs on plants with specific quantitative data (percentage increases).

NMs	Seed Germination (%)	Seedling Growth (%)	Yield (%)	References
Se	N/A	N/A	19.8%	[20]
CNTs	N/A	N/A	63%	[96]
Se	N/A	N/A	67.6%	[21]
Biochar-iron	N/A	34–200% (Shoot length), 16–118% (Root length), 5–150% (fresh biomass), 6–195% (Dry biomass)	N/A	[31]
Mn_3_O_4_	N/A	62% (Root length)	N/A	[27]
Fe_3_O_4_	N/A	27–109% (Shoot length)	N/A	[26]
ZnO-CaO	100%	N/A	N/A	[35]
Fe_3_O_4_-SiO_2_	N/A	144% (root dry weight), 243% (shoot dry weight), 34.4% (leaf area)	N/A	[37]
Zinc-Molybdenum	25%	N/A	N/A	[38]
GO	N/A	N/A	67% (fresh biomass), 65% (Dry biomass)	[44]
CuO	700%, 40%	33%, 43% (Shoot length)	N/A	[39]
TiO_2_	36%	129.27% (Shoot length), 252% (Root length), 56.10% (fresh biomass), 162.30% (Dry biomass)	N/A	[43]
Se	N/A	17% (Shoot length), 25% (leaves),	55%, 79%	[41]
ZnO	107.4%	N/A	N/A	[42]
MgO	9%	50% (Shoot length), 18% (Root length), 26% (Seedling length),	N/A	[40]

In addition to the regulation of water absorption, NMs also significantly influence energy metabolism during seed germination by enhancing starch catabolism. Experimental evidence demonstrates that Ag NMs treatment elevates α-amylase activity by 2.6-fold and doubles soluble sugar content compared to untreated controls [97]. Further examination under scanning electron microscopy (SEM) revealed that seeds treated with NMs exhibit increased starch grain density with characteristic surface pitting, indicating accelerated hydrolysis [97,98]. Additionally, NMs treatment significantly increases the hormone gibberellin acid (GA), which positively correlates with α-amylase activity and is known to induce the production of α-amylase [99]. NMs also directly upregulate the expression of alpha-amylase 1 and 2 (*AMY1* and *AMY2*), which are key genes involved in regulating α-amylase activity. Beyond transcriptional control, these genes encode proteins that physically interact with α-amylase, forming functional complexes essential for starch hydrolysis into soluble sugars [100]. These soluble sugars not only provide essential energy substrates for embryonic development but also facilitate seed germination.

Phytohormones play crucial roles in seed dormancy and germination, particularly abscisic acid (ABA) and gibberellins (GA). ABA primarily functions to prevent premature germination and induces primary dormancy, while GA promotes seed germination by antagonizing ABA-mediated inhibition [101]. NMs have been shown to promote seed germination through dynamically modulating ABA-GA balance by upregulating the expression of GA- and cytokinin (CTK)-related genes while suppressing ABA-associated genes [90,102]. For example, treatment of rapeseed with Se and ZnO NMs reduces the expression of ABA-related genes *BnCYP707A1*, *3*, and *4*, whereas it upregulates gibberellin-related genes *BnGA20ox*, *BnGA3ox*, and *BnCPS* [103]. Another study has demonstrated that priming rice seeds with Se NMs leads to an increase in GA_3_ content, accompanied by upregulation of *OsGA3ox1* and *OsGA20ox1* expression, and a decrease in ABA content, along with downregulation of *OsNCED1* expression [90,104].

NMs also modulate seed germination by influencing soil microbial communities, particularly plant growth-promoting rhizobacteria (PGPR). Take Si-based NMs as an example; they have been demonstrated to exert a positive influence on the proliferation and activity of PGPR, such as *Bacillus* spp., within the rhizosphere. This, in turn, creates a more favorable environment for seed germination [36,105]. Furthermore, the synergistic application of *Bacillus subtilis* and CuO NMs leads to a noticeable increase in germination rates and a significant boost in seedling vigor. This is evidenced by enhanced root length, shoot elongation, and total biomass accumulation when compared to single-treatment controls [106]. PGPR, which actively secrete phytohormones like IAA and enzymes that break down seed dormancy factors, work in harmony with NMs (influencing PGPR proliferation and activity) to optimize the availability of nutrients around the seeds [107]. After germination, the synergistic effect between NMs and PGPR continues. They are capable of performing nitrogen fixation and phosphorus solubilization, thereby supplying essential nutrients to the seedlings [108]. These collective findings underscore the dual functionality of the NM-PGPR synergism. It not only promotes seed germination but also facilitates post-germinative growth.

### 4.2. NMs Promote Plant Growth

NMs have been widely used in agriculture, demonstrating significant potential to enhance physiological performance, yield, and nutritional quality of vegetable crops (Table 3) [109]. The unique physicochemical properties of NMs, particularly their nanoscale dimensions and high surface area-to-volume ratio, allow targeted interactions with plant systems, thereby enhancing efficient nutrient delivery, improved bioavailability, and precision modulation of plant developmental processes.

Additionally, extensive studies have shown that NMs can significantly enhance photosynthetic efficiency, improve nutrient acquisition, and optimize plant homeostasis [110] (Figure 2c). Following NMs treatment, plants exhibited significant improvements in key photosynthetic parameters, including chlorophyll content, chlorophyll fluorescence intensity, and Hill reaction activity [111]. This enhanced photosynthetic activity directly promoted photoassimilate production, resulting in significant increases in both root and shoot biomass (fresh and dry weight), ultimately promoting vegetable growth [112,113,114,115]. Metal-based and non-metal-based NMs employ distinct mechanisms to regulate plant photosynthesis. Metal-based NMs primarily enhance photosynthesis through direct interaction with porphyrins. For example, they can modulate the magnesium-porphyrin complex in chlorophyll, which constitutes the fundamental photochemical center responsible for light harvesting and charge separation [116]. In the chlorophyll biosynthesis pathway, magnesium protoporphyrin IX monomethyl ester cyclase catalyzes the formation of the characteristic chlorin macrocycle, which is one rate-limiting step that determines chlorophyll accumulation kinetics [117]. In the chlorophyll biosynthesis pathway, magnesium protoporphyrin IX monomethyl ester cyclase catalyzes the formation of the characteristic chlorin macrocycle, which is one rate-limiting step that determines chlorophyll accumulation kinetics [118]. These metal-porphyrin complexes facilitate photosynthetic systems by accelerating electron transfer, stabilizing reaction intermediates, and enhancing enzymatic catalysis. In contrast to metal-based NMs, non-metal-based NMs impact vegetable growth and photosynthesis through diverse mechanisms. GP NMs and their derivatives have demonstrated the ability to enhance photosynthetic efficiency in vegetable crops through promoting light absorption and scattering within vegetable leaves [119], as proved by GP-based nanofibers (GFNs), which extend the spectral absorption range and increase photon capture amount, thereby elevating the total quantum yield of photosynthesis [120].

NMs can also enhance vegetable growth by modulating their nutrient uptake. For instance, SiO_2_ NMs significantly affect the absorption of vegetables to essential nutrients, particularly N, P, K, and Mg [121]. TiO_2_ NMs boost nutrient bioavailability for vegetable crops, probably by altering the soil’s physical and chemical properties [22]. Si NMs form a protective layer around vegetable roots, which improves soil structure and water retention, consequently promoting more efficient nutrient acquisition by roots [29].

NMs also precisely regulate the biosynthesis and metabolic pathways of phytohormones, maintaining their dynamic homeostasis during plant growth and development. The hormones affected by NMs include auxin (IAA), CTK, GA, ABA, brassinosteroids (BR), ethylene (ETH), salicylic acid (SA), jasmonic acid (JA), nitric oxide, and melatonin [122]. NMs can specifically regulate the expression of key phytohormone biosynthetic genes, including GA synthesis genes (*SPY*, *RGA*, *GAMYB*, *GA_3_*, and *GA_5_*), ABA synthesis genes (*NECD*, *ZEP*, and *AAO*), CTK reductase genes (*CKX1*, *CKX7*, *CKX5*, *CKX6*), BR receptor kinase genes (*BAK1*, *DET2*, and *CPD*), and ETH synthase gene (*ACS2*) [120]. NMs usually regulate phytohormones in a concentration-dependent way. High concentration of ZnO NMs inhibits the biosynthesis of CTK and IAA while promoting the synthesis of SA and ABA. However, they significantly increase the content of stress-related cytokinin cis-zeatin at moderate concentration [123]. Ag NMs have been shown to inhibit the production of ETH, which is associated with vegetable senescence [124].

Furthermore, NMs have been found to stimulate the synthesis of critical antioxidant enzymes, such as superoxide dismutase (SOD), catalase (CAT), and peroxidase (POD), which play a vital role in scavenging ROS generated under stress conditions, thereby contributing to cellular protection from oxidative damage [125]. In summary, integrated evidence demonstrates that NMs orchestrate plant homeostasis through coordinated molecular regulation of phytohormone networks and antioxidant defense systems, thereby promoting plant growth.

## 5. The Roles of NMs in Mitigating Biotic and Abiotic Stresses

### 5.1. Roles of NMs in Plant Disease Control

Plant diseases are one of the major threats to agricultural production. While traditional chemical pesticides are effective, they may lead to environmental pollution and pathogen resistance. In contrast, NMs can effectively suppress the occurrence or mitigate the damage of diseases by directly inhibiting pathogen growth, enhancing plant systemic resistance, and improving the growth environment. Therefore, NMs provide a more sustainable solution for agricultural disease control and prevention (Figure 2c).

NMs are considered to be good alternatives to conventional fungicides for plant disease control [126]. Numerous NMs exhibit direct antimicrobial activity against phytopathogens. For instance, ZnO NMs, on one hand, significantly inhibit the growth of *Fusarium oxysporum*, demonstrating an 80.73% inhibition rate in controlled greenhouse trials; on the other hand, they mitigate the severity of disease induced by *Fusarium oxysporum*, leading to enhanced yield and quality of sweet peppers [127]. Bio-Mn NMs facilitate plant defense by effectively hindering the colonization and invasive growth of bacterial wilt pathogens within watermelon roots. They inhibit the pathogen’s vegetative growth and sporulation processes, as well as distort conidial morphology, while maintaining pathogens’ cellular integrity in a compromised state [68]. Similarly, Fe_2_O_3_ NMs-B and Fe_2_O_3_ NMs-H, applied to cucumbers, effectively suppressed the occurrence of cucumber wilt disease, showing 70–75% protection efficacy compared to untreated controls [70].

NMs can also enhance plant immune responses and stress adaptation by regulating the expression of Pathogenesis-Related (*PR*) genes, modulating the content of salicylic acid (SA), triggering systemic acquired resistance (SAR), and interacting with the plant’s antioxidant system [128]. For instance, La_2_O_3_ and Bio-Mn NMs can suppress *Fusarium* wilt by activating SA signaling and SAR in watermelon [51,68]. CuO NMs significantly upregulate defense-related genes, *PR1* and *LOX1*, in cucumber plants while reducing cucumber root rot incidence under both greenhouse and field conditions [129]. In addition, CuO NMs effectively control zucchini yellow mosaic virus (ZYMV) pathogenesis in pumpkin plants. These CuO NMs upregulate the expression of antioxidant enzyme genes (*CAT* and *POX*) and *PR* genes. The resulting PR proteins block plasmodesmata after ZYMV infection, thereby preventing viral transport and systemic spread within the plants [130].

In addition to directly eliminating pathogens or inducing disease resistance, NMs can indirectly promote vegetable health and enhance disease resistance by improving their growth environment and nutrient uptake. For example, adding 10 mg/kg CeO_2_ NMs to soil not only significantly alters soil metabolites but also increases the contents of IAA, sugar molecules (such as mannose, trehalose, and sucrose), indoles, and amino acids (such as tryptophan, phenylalanine, and tyrosine) in the soil [131]. Furthermore, it greatly promotes rhizosphere metabolism related to IAA biosynthesis and enriches beneficial rhizosphere microbiomes, particularly actinomycetes. Therefore, Rhizosphere transport and local IAA synthesis promote plant growth while stimulating the production of disease-resistant compounds such as amino acids, organic acids, and flavonoids [131].

### 5.2. Roles of NMs in Plant Pest Control

Frequent pest infestations cause substantial economic losses and adversely affect agricultural production. However, conventional pesticide applications reduce soil biodiversity, diminish pollinator populations, and harm non-target organisms. Multiple studies have demonstrated the beneficial effects of engineered NMs, either as active ingredients or in nanoformulations, on pest control and plant protection [132].

NMs can rapidly eradicate pests by penetrating pest exoskeletons to disrupt their physiological structures [60]. A key mechanism involves ROS overproduction, which induces oxidative stress and cellular damage. For instance, Ag NMs compromise the antioxidant system in *Spodoptera* pest by elevating ROS levels, which cause severe cellular structural damage that ultimately leads to cell death [133]. Similarly, Se NMs induce ROS production in pest cells, causing oxidative damage to cellular components such as lipids, proteins, and DNA. Furthermore, Se NMs inhibit the activities of antioxidant enzymes, such as CAT and SOD, thereby weakening the pest’s defense system and exacerbating oxidative stress. This cascade results in mitochondrial dysfunction, energy production impairment, and eventual cell death [134]. Beyond cellular toxicity, Se NMs also interfere with the development of *Spodoptera littoralis* (*S. littoralis*), leading to lower pupation rates and shorter pupal duration [134]. Similar findings were also reported by Abd El-Latef et al., who documented reduced pupation and impaired biological parameters in *S. littoralis* following Cu and Zn NMs treatment, along with decreased feed consumption [135]. Collectively, by targeting both physiological integrity and antioxidant pathways, NMs offer a promising alternative to traditional chemical pesticides, enabling efficient pest management while reducing environmental consequences.

### 5.3. Roles of NMs in Plants Facing Abiotic Stresses

Vegetable growth is often adversely affected by abiotic stresses. Given the increase in extreme climate events, global food security will largely depend on our ability to mitigate the detrimental impacts of various abiotic stressors, including heat, drought, salinity, floods, and nutritional imbalance, on vegetable cultivation and yield [136]. Under these stress conditions, plants undergo systemic damage involving membrane disruption, organelle dysfunction, and metabolic imbalance [137]. Emerging evidence indicates that NMs can effectively alleviate these adverse effects through strengthening antioxidant defense systems, maintaining osmotic homeostasis, and triggering stress-responsive metabolic reprogramming [138] (Figure 2c). These nanoscale interventions play roles at molecular, cellular, and ecological levels to support plant physiological functions under challenging environmental conditions.

A key mechanism underlying NM-mediated abiotic stress adaptation depends on their ability to enhance endogenous antioxidant capacity. For example, Mn_3_O_4_ NMs increase endogenous antioxidant levels in cucumber leaves by upregulating precursors and downstream products in the shikimate and phenylalanine metabolic pathways [139]. Cs-Se NMs can significantly improve plant growth parameters, enhance the antioxidant defense system, improve physiological and biochemical properties, and regulate key gene expression, collectively mitigating the damage of salt stress to plants [140]. CNTs and GP NMs elevate chlorophyll, ascorbic acid, glutathione, protein, and phenol contents in vegetables, as well as increasing activities of enzymes such as CAT, APX, GPX, and PAL [66]. Beyond direct biochemical modulation, ecological interactions also contribute to stress mitigation, as demonstrated by Cs-Se NMs that can enhance systemic plant resilience by promoting beneficial rhizosphere microorganisms [140].

NMs can also enhance abiotic stress tolerance through osmotic regulation. NMs alleviate osmotic imbalance by modulating the accumulation of both organic and inorganic osmolytes. For organic solute synthesis, CaO NMs enhance flavonoid biosynthesis by upregulating key relative genes, including phenylpropanoid synthesis genes (*PAL*, *C4H*, and *4CL1*/*4CL5*), flavonoid skeleton formation genes (*CHS*, *CHI*, *F3H*, and *F3′H*), downstream modification genes (*DFR*, *ANS*, *UGT78D2*/*UGT79B1*, and *MT*), and transcriptional regulators (*PAP1/PAP2*) [141]. Similarly, TiO2 NMs alleviate PEG/Ni-induced stress by elevating proline and carbohydrate levels [142], while CMC-Nar boost drought tolerance through increased synthesis of phenolic compounds, flavonoids, and tannins [76]. Application of CaO NMs results in an increased accumulation of 28 metabolites and a decreased accumulation of 18 metabolites, primarily associated with nitrogen metabolism and amino acid biosynthesis in rapeseed. For inorganic ion homeostasis, NMs stabilize membrane integrity by regulating H^+^/K^+^ flux and Na^+^/K^+^ ratios [143], whereas Mn_3_O_4_ and CeO_2_ NMs enhance salt stress adaptation by upregulating the expression of *BnaSOS1* (salt oversensitivity 1, a sodium/hydrogen antiporter used for sodium ion exclusion) *BnaHKT1* (high-affinity sodium ion transporter), *BnaNHX1* (sodium/hydrogen exchanger used for sodium ion sequestration in the cytoplasmic matrix) to maintain cellular ion equilibrium [74].

NMs provide energy support for stress adaptation through metabolic pathway activation. Cu (OH)_2_ NMs influence central carbon metabolism by promoting glycolysis and the TCA cycle, which not only generates ATP but also supplies precursors for aromatic compound and shikimate-phenylpropanoid biosynthesis [72]. GO enhances carbon metabolism by elevating the activities of key enzymes like SPS and SS, and effectively promotes the GS-GOGAT cycle to strengthen nitrogen metabolism [144]. Nano-PM increases NADP and NADPH levels by enhancing the activities of key enzymes (NADK and G6PDH) in the pentose phosphate pathway (PPP). It is also involved in elevating mitochondrial ATPase activity, maintaining the integrity of mitochondrial membranes and structures, and regulating electron transfer during mitochondrial respiration. These collective actions facilitate the energy metabolism and alleviate senescence of plants [145].

In summary, this multilevel protective framework establishes NMs as versatile tools for abiotic stress mitigation. By targeting antioxidant systems, osmotic regulation, and metabolic networks, nanotechnology provides a comprehensive strategy to sustain vegetable productivity under increasingly challenging environmental conditions.

## 6. Challenges and Safety Considerations for NMs Applications

### 6.1. Soil Environmental Pollution and Food Safety Risks Resulting from NMs

In recent years, a large number of studies have demonstrated that NMs can enhance plants’ nutrient uptake efficiency, strengthen their resistance to pests and diseases, and increase crop yields. These advantages have driven the rapid adoption and application of NMs in agricultural practices, especially in vegetable cultivation. However, amidst these benefits, concerns have arisen regarding the potential environmental and health hazards associated with NMs use.

The plant uptake, transportation and accumulation of most nanoparticles at high concentrations cause phytotoxicity, which decreases crop productivity by modifying plants’ cellular structure, physiological and biochemical processes, and molecular irregularities [11]. Furthermore, not all NMs employed in agriculture are entirely absorbed by vegetable crops. A significant portion of them may remain in the soil [146]. It is estimated that around 9–38% of NMs from nano-products end up in soils, posing a risk to biota [147]. NMs may interact with soil components in unpredictable ways, altering soil structure, water-holding capacity, and nutrient cycling processes [148]. Take ZnO and CuO NMs as examples. These NMs can disrupt soil microorganisms by altering the synthesis of key metabolic products (such as phenazines and siderophores) in rhizosphere microbes, thereby reducing the bioavailability of iron in the soil [149].

In addition to their impacts on soil, NMs also pose threats to water bodies. The potential for NMs to enter rivers, lakes, or groundwater through runoff or leaching from agricultural fields further heightens environmental worries.

The environmental pollution caused by NMs in soil also raises concerns about food safety. Several studies have indicated that NMs may enter the human body through the food chain and have potential health effects [150]. For example, NMs may penetrate alveolar and cellular membranes, directly affecting the respiratory and immune systems of animals. Inhalation of carbon nanotubes can lead to lung tissue damage and lung fibrosis. Inhalation of carbon nanotubes may lead to lung cancer in the same way that inhalation of asbestos fibers does [151].

Besides respiratory and lung-related issues, NMs can also cause other health problems. Additionally, research has pointed out that nanoplastics are capable of causing the breakdown of physiological balance in the human body, resulting in blood clot formation or the occurrence of cardiovascular diseases [152]. Given these findings, it is imperative to conduct adequate risk assessments when using NMs in food crops to ensure their safety for human consumption, as neglecting these aspects could undermine the very benefits that NMs aim to provide [125].

### 6.2. Methods for Assessing the Safety Performance of NMs

With the increasing application of engineered nanoparticles in electronics, consumer products, pesticides, food, and pharmaceutical industries, concerns are growing about their potential human health hazards [153]. Thus, there is an urgent need for rapid and reliable methods to assess the health hazard potential of engineered NMs [154].

Traditional methods for evaluating the safety of NMs are not only time-consuming and costly but also resource-intensive and raise ethical concerns due to their dependence on animal testing. Following the 3Rs principle (reduction, refinement, and replacement of animal use in research), there is a pressing need to develop alternative strategies for in vitro testing of NMs [155]. As a result, an increasing number of models, testing methodologies, and machine learning-based approaches for NM safety assessment are emerging.

To examine NMs in vitro, various models and tests can be used, including cell culture models and physicochemical analyses of core–shell-coating interactions. Human-derived cells (either cell lines or primary cells) maintained under physiologically relevant, in vivo-mimicking conditions may serve as an optimal model system. Beyond standard toxicity testing parameters, special consideration should be given to emerging toxic effects, particularly those resulting from epigenetic modifications [7]. By establishing innovative testing strategies, we can advance toward a more efficient, ethically sound, and comprehensive framework for NMs safety assessment.

Furthermore, researchers have developed a machine learning-based approach to predict the toxic effects of NMs on cells. Using automated optimization tools and synthetic data augmentation techniques, a study addresses class imbalance issues through parameter optimization and synthetic data generation [156]. Additionally, it develops a domain-specific applicability assessment framework for nanotoxicology predictions, significantly improving the reliability of model outputs. This approach establishes an efficient and accurate computational framework for NMs safety assessment, highlighting the significance of interdisciplinary collaboration in accelerating nanotechnology innovation and promoting environmentally sustainable nanotechnology development [157].

The safety performance assessment of NMs constitutes a multifaceted challenge that requires the application of rigorous scientific methodologies and analytical tools to fully characterize their potential hazards. Such comprehensive evaluations provide a solid scientific basis for formulating effective regulatory strategies.

## 7. Conclusions and Outlook

With the ongoing technological advancements in NMs development, NMs are expected to be more widely used in agricultural production systems. This widespread application is anticipated to not only enhance agronomic efficiency, such as by improving crop yields and quality, but also align with sustainable agricultural practices by reducing the reliance on chemical fertilizers and pesticides, thus minimizing environmental pollution.

Existing studies have demonstrated the effects of NMs on vegetable growth and development, their underlying mechanisms remain incompletely understood. Additionally, the potential of NMs in mitigating stress damage requires further exploration. Current applications of NMs in stressed plants mainly target the induction of plant defense responses. Nevertheless, emerging evidence suggests that NMs may also enhance vegetable stress resistance through alternative mechanisms, such as by modulating both rhizospheric and endophytic microbial communities. In the future, integrating multi-omics technologies, including genomics, proteomics, and metabolomics, will enable us to gain a deeper understanding of the roles of NMs in modulating vegetable cultivation.

It is important to note that different vegetable species or varieties may exhibit significant variability in their responses to NMs. For example, certain NMs (e.g., CNTs, Se, etc.) may be more effective in promoting the growth of leafy vegetables like spinach and lettuce, potentially by enhancing chlorophyll synthesis and photosynthetic efficiency. Conversely, other NMs (e.g., ZnO, etc.) may show greater efficacy in root vegetables such as carrots and radishes, possibly by stimulating root elongation and nutrient absorption. Additionally, the optimal concentration of NMs can vary considerably among vegetable crops; low doses may be insufficient to elicit beneficial effects, while excessive concentrations could induce toxicity, leading to stunted growth or tissue damage. Despite these variations in response, systematic approaches can help identify suitable NM types and concentrations. For instance, by considering the specific needs and characteristics of each vegetable species or variety, conducting preliminary trials to determine the potentially optimal dosage and application method, and selecting NM types that are biologically compatible with the target vegetables, we can effectively manage this variability and ensure safer, more efficient use of NMs in agriculture.

Precision application of NMs will thus emerge as a pivotal research area. Current methods of nanoparticle application are still relatively coarse. Future development should focus on engineering intelligent NMs capable of targeted translocation and controlled release within vegetable tissues, thereby improving utilization efficiency while minimizing negative impacts on the environment and ecology. Concurrently, rigorous safety assessment and risk prevention of NMs must be prioritized. This necessitates establishing a multidisciplinary safety evaluation framework to systematically assess the influences of NMs application on environments, enabling science-based risk management protocols that ensure sustainable implementation of NM technologies in agriculture while safeguarding ecosystem integrity.

## Figures and Tables

**Table 1:** Nanofertilizers applied in vegetable cultivation.

Nanofertilizers	Concentration	Size (nm)	Vegetable Species	References
Se	0.5 mg/kg	62.3 ± 14.6 nm	Chinese cabbage	[20]
Se	10 mg/L	61.9 ± 13.7 nm	Cherry radish	[21]
Biochar-iron	500 mg/kg	N/A	Chinese cabbage	[31]
Mn_3_O_4_	10 mg/kg	104.1 mm	Radish	[27]
Fe_3_O_4_	10 mg/L	N/A	Coriander	[26]
CeO_2_	50 mg/kg	7.0 nm	Carrot	[19]
Mn_0.5_Zn_0.5_Fe_2_O_4_	100 mg/L, 200 mg/L	14 nm	Squash	[32]
NaYF_4_:Yb,Er@CDs	0.5 mg/mL	N/A	Mung bean	[33]
TiO_2_	100 mg/L	5 nm	Tomato	[22]
TiO_2_	20–200 μg/mL	80 ± 15 nm	Red bean	[23]
Fe	N/A	2–6 nm	Mung bean	[34]
ZnO-CaO	500 ppm	N/A	Mung bean	[35]
Graphene	1000 mg/L	8–12 nm	Tomato	[24]
Si	100 mg/L, 1000 mg/L	10–17 nm, 110–120 nm	Tomato	[36]
Fe_3_O_4_-SiO_2_	100 mg/L	N/A	Spinach	[37]
ZnMo	124–10 mg/L	200 nm	Pepper	[38]
CuO	10 mg/L	23.43 nm	Lettuce, Tomato	[39]
MgO	100 mg/L	15–20 nm	Green gram	[40]
Se	N/A	150 nm	Potato	[41]
ZnO	800 mg/L	20–60 nm	*Allium cepa* L.	[42]
TiO_2_	10 μm	12.8 nm	Faba bean	[43]
GO (Graphene oxide)	1200 mg/L	10–100,000 nm	Mung bean	[44]

## Data Availability

No new data were created or analyzed in this study. Data sharing is not applicable to this article.
